# Neuroimaging in psychiatry: is it relevant?

**DOI:** 10.1192/j.eurpsy.2022.1646

**Published:** 2022-09-01

**Authors:** I. Binic, J. Petrovic, J. Antonijevic, O. Zikic, D. Pancic, F. Petrovic

**Affiliations:** 1 Clinical Center Nis, Psychiatry, Nis, Serbia; 2 Special Psychiatric Hospital “Gornja Toponica”, Psychiatry, Nis, Serbia; 3 Medical Faculty Nis, Psychiatry, Nis, Serbia; 4 University of Nis, Medical Faculty, Clinical Center Nis, Psychiatry, Nis, Serbia; 5 Medical Faculty Nis, Forensic Medicine, Nis, Serbia; 6 Clinical Center Nis, Radiology, Nis, Serbia

**Keywords:** Neuroimaging, psychiatry

## Abstract

**Introduction:**

The upturn of neuroimaging techniques in the past 30 years has changed the study of the biology of psychiatric disorders with implications for psychiatric practice. Thrive in medical imaging technology has, in fact, truly reformed nearly every medical field.

**Objectives:**

These advances include both improvements in image resolution and the development of novel imaging techniques all of which provide an unprecedented view, in detail, of anatomical structures and/or functions in the human body.

**Methods:**

Nowadays, we are familiar with the role of some brain structures such as the amygdala, the thalamus, the hippocampus, the dorsolateral prefrontal cortex, and the insula in neuropsychiatric function. For example, lesions to the frontal cortex can disrupt judgment, motivation and social behavior.

**Results:**

Currently, most imaging techniques have some sort of clinical application, but this is usually restricted to a limited number of cases. New techniques have provided invaluable information not only about the brain structure and function associated with psychiatric disorders but increasingly about the mechanisms underpinning these disorders.

**Conclusions:**

Growing understanding of the specific pathophysiology of mental disorders prepares us for improvement in the identification of diagnostic and prognostic biomarkers, which could lead to more accurate diagnoses and prediction of treatment response of the disorders managed in everyday clinical practice. Of note, the identification of neural biomarkers could potentially identify people at risk of developing a particular illness.

**Disclosure:**

No significant relationships.

